# Hypoxia-Inducible Factors Regulate Osteoclasts in Health and Disease

**DOI:** 10.3389/fcell.2021.658893

**Published:** 2021-03-18

**Authors:** Xianyi Meng, Ben Wielockx, Martina Rauner, Aline Bozec

**Affiliations:** ^1^Department of Internal Medicine 3 – Rheumatology and Immunology, Friedrich-Alexander-University Erlangen-Nurnberg (FAU) and Universitatsklinikum Erlangen, Erlangen, Germany; ^2^Institute of Clinical Chemistry and Laboratory Medicine, Technische Universität Dresden, Dresden, Germany; ^3^Department of Medicine 3 – Division of Molecular Bone Biology, Medical Faculty of the Technische Universität Dresden, Dresden, Germany

**Keywords:** hypoxia, HIF, osteoclast, osteoblast, bone homeostasis, inflammation, malignant bone disease

## Abstract

Hypoxia-inducible factors (HIFs) have become key transcriptional regulators of metabolism, angiogenesis, erythropoiesis, proliferation, inflammation and metastases. HIFs are tightly regulated by the tissue microenvironment. Under the influence of the hypoxic milieu, HIF proteins allow the tissue to adapt its response. This is especially critical for bone, as it constitutes a highly hypoxic environment. As such, bone structure and turnover are strongly influenced by the modulation of oxygen availability and HIFs. Both, bone forming osteoblasts and bone resorbing osteoclasts are targeted by HIFs and modulators of oxygen tension. Experimental and clinical data have delineated the importance of HIF responses in different osteoclast-mediated pathologies. This review will focus on the influence of HIF expression on the regulation of osteoclasts in homeostasis as well as during inflammatory and malignant bone diseases.

## Introduction

Bone is a highly dynamic tissue that undergoes constant remodeling to adapt to changing functional and metabolic demands, but also to repair microdamages that naturally occur throughout life. For example, bone sensitively reacts to loading (e.g., weight-lifting activities) or unloading conditions (e.g., space flight) by increasing or decreasing bone mass, respectively. In addition, bone also adapts to meet changing metabolic demands (Zhang et al., 2015; Shahi et al., 2017; Loeffler et al., 2018), such as during lactation, when bone resorption increases to provide sufficient calcium for milk production (Kovacs, 2005). Thus, bone remodeling is a finely tuned and dynamic system that is required to maintain bone mass as well as mineral homeostasis during adulthood (Al-Bari and Al Mamun, 2020).

Bone remodeling is a temporally and spatially controlled process (Frost, 1963). In adults, about 10% of the bone surface is undergoing remodeling at a given time. The cells that contribute to bone remodeling are grouped into the basic multicellular unit (BMU) (Hauge et al., 2001; Andersen et al., 2009). Therein, osteoclasts, which are of hematopoietic origin, resorb bone (Teitelbaum, 2000). This is followed by a reversal phase, in which osteoclasts vacate the bone remodeling area and allow for osteoblasts, the bone-forming cells, to locate and refill the resorbed area with new bone matrix (Matsuo and Irie, 2008; Sims and Gooi, 2008). This process is estimated to take about 3 months in humans (Eriksen et al., 1984a,b). It is a coupled process, where osteoclasts regulate the differentiation and activity of osteoblasts and vice versa. Besides osteoclasts and osteoblasts, which are the two most important specialized cell types for bone remodeling, several other cell types have been shown to contribute to bone remodeling, such as the osteocytes, which appear to coordinate bone remodeling by sending signals to the osteoclasts and osteoblasts to regulate their activity (Sims and Martin, 2015).

In healthy adults, the amount of newly formed bone equals the amount of resorbed bone, thus, ensuring the maintenance of bone mass. However, several disease conditions including estrogen deficiency, chronic inflammation, and malignant disease lead to uncoupling of bone resorption and bone formation in which bone resorption exceeds bone formation, leading to bone loss and fragility (Roodman, 2004; Pacifici, 2008; Redlich and Smolen, 2012; Klein-Nulend et al., 2015). As such, osteoclasts play a prominent role in diseases characterized by bone loss and therefore are the main therapeutic target of anti-resorptive strategies to treat osteoporosis.

Importantly, both, inflammation and malignancy are characterized by hypoxia and also physiological bone remodeling is under the strict control of hypoxia-related signaling pathways. The latter may be explained by the rather hypoxic microenvironment of bone niche, with *in vivo* measurements in mice demonstrating local oxygen tension as low as 1.3 kPa (10 mmHg; tissues less than this are generally defined as hypoxic) (McNamee et al., 2013; Spencer et al., 2014).

**FIGURE 1:**
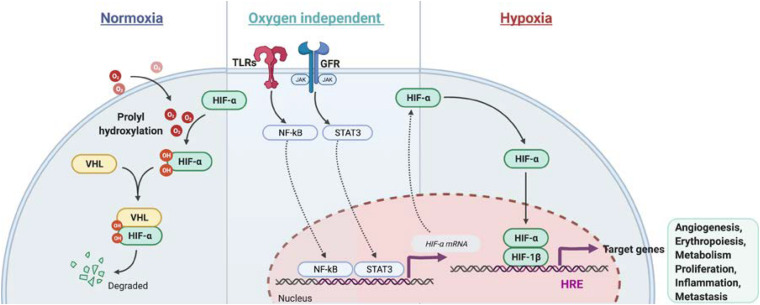
Regulation of HIFα stabilization in normoxia and hypoxia conditions as well in an oxygen independent pathway. In normoxia, HIFα is hydroxylated by prolyl hydroxylase domain (PHD) protein. The proline-hydroxylated HIFα is then recognized by von Hippel-Lindau E3 ubiquitin ligase (VHL) and subjected to be degraded *via* proteasome. In hypoxia, HIFα becomes stable and dimeries with HIF-1β. After translocation to the nucleus, the HIF heterodimer binds the hypoxia response element (HRE) of target genes to regulate transcription. Besides, NF-κb and STAT3 signaling activation induce *Hifa* mRNA transcription under normoxia or hypoxia.

Hypoxia-inducible factors (HIF) are heterodimeric transcription factors, consisting of an oxygen-labile alpha subunit (HIFα) and a constitutively-stable beta subunit (HIF1β), that exert pivotal roles in inducing cellular responses to hypoxia (Wang et al., 1995; Tian et al., 1997). HIF1α and HIF2α are structurally similar (Loboda et al., 2010). Their stability is post-transcriptionally regulated by oxygen availability through the iron-dependent enzymes prolylhydroxylases (PHDs) (Mole, 2010). In well-oxygenated environment, HIFα is subject to oxygen-dependent hydroxylation at proline residues 564 and/or 402 by PHDs, which leads to binding of the von Hippel Lindau protein (VHL) and an associated ubiquitin protein ligase complex. This leads to ubiquitination and proteasomal degradation of HIFα (Lee et al., 2004). Conversely, the hydroxylation reaction is inhibited under hypoxic condition, HIFα subunits are stabilized and translocate to the nucleus, where they heterodimerize with HIF1β and bind to HRE located within regulatory elements of HIF target genes (Dengler et al., 2014). These are involved in multiple processes such as angiogenesis (*Vegf*, *Pdgf*, and *Fgf2*), erythropoiesis (*Epo*, *Tfr1*, and *Cp*), metabolism (*Glut1*, *Pdk1*, *Hk2*, *Ldha*, and *Mct4*), proliferation (*Tnfa*, *Ccnd1*, and *Igf2*), inflammation (*Il1b*, *Il6*, and *Il17*) and metastasis (*Met1*, *Lox1*) (Flamme et al., 1997; Jaakkola et al., 2001; Mahon et al., 2001; Wenger et al., 2005; Semenza, 2014). Recent studies showed that many other proteins are involved in the regulation of basal HIF1α levels in an oxygen-independent manner. Luo et al. (2010) delineated that the heat shock protein 70 (HSP70) binds *via* its carboxy-terminus to HIF1α, leading to recruitment of HSP70-interaction protein (CHIP), a chaperone-dependent E3 ubiquitin ligase, which mediates HIF1α ubiquitination and proteasomal degradation. Additionally, it has been shown that PTEN-PI3K-AKT signaling axis controls E3 ubiquitin-protein ligase Murine double minute 2 (MDM2), which mediates HIF1α ubiquitination under hypoxic conditions in a proteasome-dependent manner (Joshi et al., 2014; Figure 1).

Besides the post-transcriptional regulation of HIF1α protein stability, HIFα is also regulated at the transcriptional level. Increased transcription of *Hif1a* was found in cells after the stimulation of growth factors (FGF, EGF, and Heregulin), cytokines (TNF-α, IL-1, and IL-6) and pathogen associated molecular patterns (PAMP) (LPS and HBx) *via* the JAK/STAT and NF-κB signaling pathways (Frede et al., 2006; Figure 1). Recognition of pathogens by immune cells activates the mitogen activated protein kinase (MAPK) pathway *via* pattern recognition receptors signaling, such as toll like receptors (TLRs) (Frede et al., 2006), which leads to the induction of NF-κB and transactivation of *Hif1a* under normoxia (Rius et al., 2008). In addition, T cell receptor ligation induces substantial accumulation of HIF1α mRNA and protein, especially in the pro-inflammatory T helper 17 (Th17) cell lineage by a mechanism dependent of STAT3 signaling activation (Dang et al., 2011). Besides, MYD88-dependent NF-κB activity is crucial for LPS-induced HIF1α accumulation in dendritic cells (Jantsch et al., 2011). Taken together, growth factors, cytokines and factors stimulating PAMP are critical regulators of HIF1α or HIF2α level in normoxic and hypoxic conditions.

Given the pertinent role of osteoclasts in bone homeostasis and bone disease, and their regulation *via* hypoxia signaling, this review will summarize the current knowledge on the role of hypoxia signaling on osteoclasts and its potential as therapeutic target to inhibit osteoclast function in inflammatory and malignant bone diseases.

## Osteoclasts and Their Regulation by Hypoxic Signaling Pathways

Osteoclasts originate from the erythromyeloid progenitors during embryogenesis and throughout life fuse with hematopoietic stem cells to produce long-lived, multinucleated cells that are capable to resorb bone (Jacome-Galarza et al., 2019). Mononuclear cells also appear to dissociate again from multinucleated osteoclasts, suggesting that osteoclasts are undergoing constant remodeling themselves. Receptor activator of NF-κB ligand (RANKL) is the key cytokine driving osteoclastogenesis. Upon binding to its receptor RANK, RANKL induces differentiation, fusion, and life span of osteoclasts *via* the activation of pathways downstream of TRAF6 including MAPK, NF-κB, and PI3K/Akt (Nakagawa et al., 1998; Kong et al., 1999; Yahara et al., 2020). These pathways culminate in the activation of NFATc1, AP-1, and NF-κB transcription factors, which induce the expression of typical osteoclastic genes such as cathepsin K or tartrate-resistant acid phosphatase (TRAP). RANKL is mainly produced by cells from the osteogenic lineage (i.e., osteoblasts and osteocytes) together with its natural antagonist osteoprotegerin (OPG). OPG is able to bind RANKL and prevent it from binding to RANK and thus initiating osteoclastogenesis. Therefore, the RANKL/OPG ratio is crucial for predicting the osteoclastic milieu of the environment. Once osteoclasts are formed they attach tightly to the bone matrix *via* integrins, most prominently α_*v*_β_3_, and seal off the environment from the area that will be resorbed. Within this sealing zone, osteoclasts acidify the environment and secrete matrix-degrading enzymes such as cathepsin K into the resorption lacunae to resorb the mineralized and organic components of bone. Until recently, osteoclasts have been proposed to undergo apoptosis after bone resorption. However, newer concepts suggest that osteoclasts may recycle (parts) of themselves to fuse with new osteoclast syncytia and engage in new remodeling cycles (McDonald et al., 2019).

Hypoxia is a critical stimulator of osteoclastogenesis in mouse and human cell culture systems. Early studies have shown that low oxygen tension (2% O_2_) increases osteoclast differentiation and bone resorption *in vitro* (Arnett et al., 2003; Muzylak et al., 2006; Bozec et al., 2008), while hyperoxia suppresses osteoclastogenesis (Al Hadi et al., 2013; Yu et al., 2020). In an effort to analyze whether HIF1α was responsible for the pro-osteoclast effects, HIF1α protein was expressed in osteoclasts *in vitro* (Leger et al., 2010). However, in this study, osteoclast generation was inhibited by expression of a constitutively active form of HIF1α, suggesting that other hypoxia-responsive factors may contribute to osteoclastogenesis (Leger et al., 2010). Another study that investigated the potential of hypoxia mimetic PHD inhibitor dimethyloxallyl glycine (DMOG) to rescue ovariectomy-induced bone loss similarly found no effect of DMOG on osteoclast activity (Peng et al., 2014). Finally, Hulley et al. (2017) have shown that activation of HIF1α *via* deficiency of PHD2 does not affect osteoclast differentiation, but impairs bone resorption *in vitro*, suggesting that HIF1α may affect the bone resorbing activity of osteoclasts. However, it should be noted that *in vivo*, PHD2 +/− mice showed normal serum levels of CTX, a bone resorption marker, despite low bone mass (Rauner et al., 2016), suggesting that rather defective osteoblast function contributed to the low bone mass. HIFs are the canonical substrates for PHD-mediated protein hydroxylation. Increasing *in vitro* evidence indicates that PHD may also have alternative targets such as IKK-β, p105, p53, and FOXO3a (Cockman et al., 2019; Lee, 2019). However, the role of these non-HIF substrates in osteoclastogenesis under hypoxia is still elusive. Besides, neither osteoclast-specific knockout nor treatment with a HIF1α inhibitor altered bone mass or osteoclast numbers under physiological conditions, but in states of estrogen or testosterone deficiency, when osteoclasts are activated, HIF1α deficiency prevented bone loss by suppressing osteoclast activation (Miyauchi et al., 2013; Tando et al., 2016). Overall, direct effects of HIF1α on osteoclastogenesis during physiology appear negligible. However, metabolically, osteoclasts require oxidative phosphorylation during differentiation, while for bone resorption, osteoclasts rely on energy production *via* glycolysis (Czupalla et al., 2005; Jin et al., 2014; Lemma et al., 2016). As HIFs activate glycolysis, this may in part explain the stronger effect of HIF1α activation on osteoclastic bone resorption rather than differentiation. In fact, HIF1α has been identified as a critical metabolic switch to turn on anaerobic respiration to rapidly increase ATP production in osteoclasts (Morten et al., 2013).

Importantly, there is strong evidence that HIF1α controls osteoclastogenesis *via* the regulation of the RANKL/OPG ratio and also IL-33 levels in osteoblasts (Wu et al., 2015; Kang et al., 2017; Zhu et al., 2019) and osteocytes even under physiological conditions (Stegen et al., 2018).

In contrast to HIF1α, HIF2α appears to have direct effects on osteoclasts. Overexpression of HIF2α in progenitors increased osteoclast numbers and marker gene expression *in vitro* (Lee et al., 2019) by upregulating TRAF6 expression. Moreover, osteoclast-specific knockout of HIF2α increased bone mass by decreasing osteoclast numbers (Lee et al., 2019). However, also in case of HIF2α, osteoblast-mediated regulation of osteoclastogenesis *via* RANKL/OPG seems to play an important role as osteoblast-specific knockout of HIF2α also decreased osteoclast numbers *in vivo.*

Taken together, HIFs appear to play a more prominent role in osteoblast-to-osteoclast communication rather than directly affecting osteoclastogenesis. Moreover, activation of hypoxia signaling pathways may be more relevant in disease states than during physiological bone remodeling. In the following sections, we will discuss the role of hypoxia-related proteins in inflammatory and malignant diseases.

## Role of Hypoxia in the Regulation of Osteoclasts in Rheumatoid Arthritis as a Prototypical Inflammatory Disease

**FIGURE 2:**
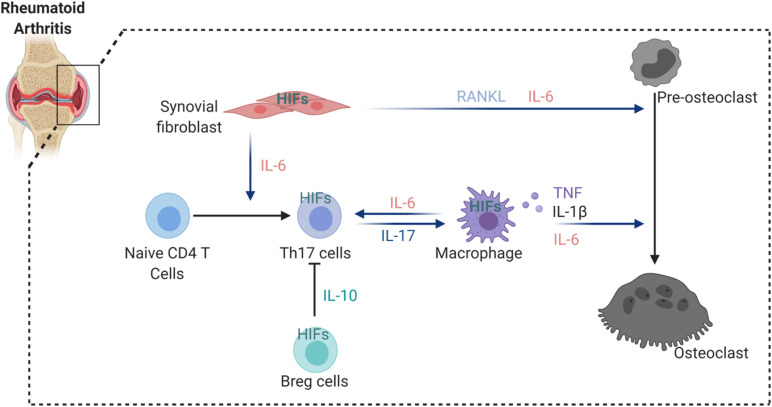
HIFs involvement in the regulation of osteoclastogenesis in arthritic joint. HIFs expression induce the secretion of a number of cytokines from synovial fibroblast and macrophages that enhance osteoclastogenesis. In addition, hypoxia stimulates IL-6 production by synovial fibroblast and macrophages, thereby increasing Th17 differentiation from naïve CD4 T cells. However, HIFs activation in Breg cells inhibit Th17 differentiation *via* suppressive factor IL-10.

Rheumatoid arthritis (RA) is a systemic autoimmune disorder that manifests as chronic inflammation and joint tissue destruction (Komatsu and Takayanagi, 2012). Macrophages, T lymphocytes and B lymphocytes are crucial cells in the development and progression of RA (Ma and Pope, 2005; Cope et al., 2007; Marston et al., 2010). Oxygen tension in the synovial fluid of RA patients (range from 18 to 33 mmHg, equivalent to 2 to 4%) was found lower than in healthy controls (range from 69 to 89 mmHg, equivalent to 9 to 12%) (Giatromanolaki et al., 2003; Muz et al., 2009). In addition, tissue oximeters were used to confirm that hypoxia is a feature of RA synovial tissue and correlates with the intensity of the inflammatory process during RA development (Quinonez-Flores et al., 2016). HIFs (HIF1α and HIF2α) could therefore interfere with joint inflammation, angiogenesis and cartilage destruction in RA (Westra et al., 2010; Quinonez-Flores et al., 2016). Different aspects of RA are influenced by the expression of HIFs in stromal cells and immune cells. Hypoxia induces vascular cell adhesion molecule-1 (VCAM1) and stromal cell-derived factor-1 (SDF-1) expression in synovial fibroblasts and promotes lymphocyte homing to joints of RA patients (Hitchon et al., 2002; Hu et al., 2016). Studies have also shown that NF-κB-HIF1α pathway activation drives the migration and invasion of synovial fibroblasts by increasing the expression of MMP2 and MMP9 (Lee et al., 2012). Interestingly, HIF2α was expressed mainly in fibroblast-like synoviocytes (FLS) of RA synovium and regulated the production of RANKL and several catabolic factors such as matrix-degrading enzymes (MMP3, MMP9, MMP12, MMP13, and ADAMTS4), chemokines (CCL2, CCL5, CCL7, CXCL1, CXCL2, CXCL4, CXCL5, and CXCL10) and inflammatory mediators (COX2 and iNOS) (Huh et al., 2015). Moreover, HIF2α expression in FLS controls IL-6 induction and enhances Th17 cell differentiation during RA pathogenesis (Ryu et al., 2014). Also the induction of IL-1, TNF-α, and IL-33 was reported to be increased in FLS *via* HIFs, which subsequently was reflected by T cell functions with expansion of Th1 and Th17 cells (Sarkar et al., 2010; Samarpita et al., 2018), but also B cell autoantibody production. By a feedback loop by TNF-α, IL-33 manages the control of HIF. Besides HIF2α, also HIF1α increases IL-6 production in RA. Further evidence shows that HIF1α is highly expressed in Th17 cells and that loss of HIF1α in Th17 cells impairs their differentiation and IL-17 production, suggesting that HIF1α expression in Th17 cells might control synovial inflammation in arthritis (Dang et al., 2011). Finally, HIF1α participation in collagen-induced arthritis has been demonstrated by studying conditional HIF1α deletion in B cells, which results in less IL-10-producing B cells and exacerbated Th17 cells mediated inflammation (Meng et al., 2018; Figure 2).

Altogether, HIF1α and HIF2α indirectly regulate osteoclast-induced bone erosion through the control of the pro-inflammatory milieu. However, it remains unclear whether there is a direct involvement of these factors in osteoclasts under inflammatory conditions. Several therapeutic agents use the hypoxic milieu to get activated and to deliver the therapeutic agents to hypoxic cells on a site specific. However, the off-target effects might be an important challenge since hypoxic conditions also appear physiologically (Phillips, 2016). Several agents targeting the HIF pathway, such as specific HIF inhibitors, showed promising results in cancers or in hypoxia-related diseases (Fallah and Rini, 2019). Regarding RA, it has been suggested that local administration of these compounds could avoid their early systemic degradation. Another promising route may be achieved *via* delivery carriers for example *via* the delivery of gene therapy targeting HIFs. However, before these therapies approach clinics, several challenges still need to be addressed. In RA, downstream targets of HIFs have been therapeutically targeted, such as antibodies against VEGF, or small molecules against its receptor. In preclinical studies, these approaches showed a significant reduction of inflammation, particularly in the early phase of inflammatory RA development (Lu et al., 2000; De Bandt et al., 2003; Maruotti et al., 2014). Based on the above-mentioned studies, it is evident that HIFs are promising targets for RA.

## Role of Hypoxia in the Regulation of Osteoclasts in Osteolytic Bone Disease

Bone metastases are incurable, cause pathological fractures, hypercalcemia and reduce the quality of life (Macedo et al., 2017). Initially, the hypoxic bone microenvironment provides an excellent soil for tumor cells to thrive. Once homed, these cells start producing a variety of cytokines and growth factors that activate cells, including osteoclasts (Maurizi and Rucci, 2018). In turn, this will lead to bone absorption and destruction of the microenvironment, which eventually stimulates the proliferation of tumor cells. As such a vicious cycle between tumor cells and osteoclasts is established (Huang et al., 2020). Although osteolytic lesions have been observed in several cancer types, it has been especially detected in breast cancers; a tumor with great avidity for bone metastasizing in up to 80% of stage IV breast cancer patients (Brook et al., 2018). These tumor cells typically produce parathyroid hormone related protein (PTHrP), which stimulates calcium release from bone (Poole and Reeve, 2005), just like PTH. Interestingly, the expression of PTHrP is driven by HIF2α, but not HIF1α, and is not only confined to metastatic tumor cells, but has also been detected in chondrocytes where it is also induced by HIF1α (Browe et al., 2019). PTHrP stimulates osteoblasts to generate RANKL, simultaneously preventing OPG production (Huang et al., 2004). RANKL and RANK have also been shown to be produced by tumor cells in a HIF1α-dependent manner (Tang et al., 2011), suggesting that osteolysis is potentially also feasible through an interaction between the tumor cells and osteoclasts. As mentioned before, in this setting, osteoclastogenesis is driven forward due to an enhanced RANKL/OPG ratio. The consequence is enhanced bone resorption and release of other growth factors including TGFα and PDGF, both stimulating tumor growth and eventually also osteolysis (Janssens et al., 2005). Nevertheless, there are also other reports suggesting RANKL is not HIF-dependent. In that respect, deletion of PHD2 and PHD3 in osteoblast (progenitors) (Osx:cre line) resulted in increased bone volume as a consequence of OPG induction, whereas RANKL levels were not changed (Wu et al., 2015). This finding was also confirmed using a VHL knock-out strategy in primary osteoblasts (Shao et al., 2015) and by us, showing that PHD2 deletion in osteoblasts (Osx:cre line) causes high bone density (Rauner et al., 2016; Stegen et al., 2016). Although more research will be necessary to unravel the background of these opposing results, hypoxia and hypoxia pathway proteins have an impact on stromal cells of the bone/bone marrow environment that directly regulate bone homeostasis and therefore probably also osteolytic lesions.

Interestingly, the impact of hypoxia signaling in bone can also influence the growth and dissemination of external tumors finally ending up in the bone. Devignes and colleagues elegantly showed that HIF-induced CXCL12 production in osteoblast progenitors directly promotes systemic tumor growth and dissemination. In fact, mice conditionally deficient for HIF1α in osteoprogenitors displayed reduced CXCL12^+^ cells whereas VHL deficiency resulted in the opposite outcome (Devignes et al., 2018). The chemokine CXCL12/stromal cell-derived factor 1 alpha (SDF1) has not only been shown in a variety of different tumor types (Shi et al., 2020), but also plays a central role in the bone marrow niche where it controls hematopoietic stem cell quiescence in conjunction with its receptor CXCR4. In the context of breast cancer cell dissemination, this signaling appears to work *via* CXCR4 on the tumor cells, underscoring local hypoxic signaling in the BM niche exerting control on distant tumors, impacting growth and metastasis (Xu et al., 2015). This suggests that targeting CXCL12/CXCR4 would be beneficial, but different experimental approaches reveal case-by-case differences (Zielinska and Katanaev, 2020). Indeed, although systemic CXCR4 inhibition might be beneficial in breast cancer growth, deficiency of CXCR4 in osteoclasts was shown to enhance osteoclastogenesis, which in turn may again promote bone metastasis and stimulate the vicious cycle (Zielinska and Katanaev, 2020).

Conversely, hypoxia in a variety of different tumors outside the bone/bone marrow area can also affect the bone and its environment by enhancing future colonization of tumor cells and even promoting pre-osteolysis. First indications for this paradigm were reported almost two decades ago, as researchers found a clear correlation between HIF1α expression in primary breast cancers and the presence of (micro)metastasis in the bone marrow of these patients (Woelfle et al., 2003). HIF1α-induced lysyl oxidase (LOX), a copper-dependent amine oxidases, is such a molecule that can cause tumor cell dissemination and tumor driven osteolytic lesions (Bondareva et al., 2009). At the same time, it promotes RANKL-dependent differentiation of osteoclasts, while inhibiting osteoblast differentiation (Reynaud et al., 2017). This suggests that LOX secreted by tumor cells induces osteoclastogenesis thereby creating a pre-metastatic niche that would favor tumor homing and growth. Interestingly, LOX induction itself also enhances HIF1α expression, underscoring the synergism between LOX/HIF in regulating the adaptation of tumor cells to hypoxia (Pez et al., 2011) and beyond.

## Conclusion

The role of hypoxia and HIFs is evident in bone physiology and in numerous pathophysiological diseases where osteoclasts are activated and induce bone loss. However, the exact role of HIF1α or HIF2α in osteoclast remains quite vague and largely appear to be mediated indirectly *via* other cells like stromal cells. However, they should be taken into consideration when thinking of the indirect pathway of osteoclast activation, notably by their function in the immune cells, in particular in Th17/Treg cells or in macrophages. Therefore, HIF inhibitors would likely target osteoclast activation and secondary bone loss in numerous diseases. As example, a recent study discovered an increased bone mass in mice treated with HIF1α inhibitor 2ME2 (Miyauchi et al., 2013). However, it still remains inconclusive whether HIF inhibitors would act the same way in human bone diseases as in murine models. Future studies on HIF signaling and its clinical relevance may improve our understanding of the role of HIF in osteoclastogenesis and eventually lead to effective treatments for human diseases involving bone homeostasis.

## Author Contributions

MR and AB contributed equally to the work. XM, BW, MR, and AB conceived and wrote the review. All authors contributed to the article and approved the submitted version.

## Conflict of Interest

The authors declare that the research was conducted in the absence of any commercial or financial relationships that could be construed as a potential conflict of interest.
